# Whose time is a body allowed? Virtual reality, aging, and temporal justice

**DOI:** 10.3389/fpubh.2026.1948941

**Published:** 2026-09-21

**Authors:** André Ramalho

**Affiliations:** 1 Department of Sport and Well Being, Polytechnic University of Castelo Branco, Castelo Branco, Portugal; 2 SPRINT Sport Physical Activity and Health Research & Innovation Center, Polytechnic University of Castelo Branco, Castelo Branco, Portugal

**Keywords:** older adults, physical activity, tempo fit, temporal justice, virtual reality

## Abstract

Virtual reality is increasingly being used to support physical activity, rehabilitation, and participation in later life. Virtual reality-based interventions also shape the temporal conditions of participation through pacing, interruption, progression, recovery, and support requirements. *Temporal justice* offers a way to examine these conditions through three related dimensions: temporal autonomy; the fit between system rhythms and changing capacities; and the distribution of temporal authority and time-related work among older adults, caregivers, practitioners, and institutions. Temporal demands become justice-relevant when they are insufficiently justified or disproportionate, when foreseeable differences in capacity cannot reasonably be accommodated, or when the work required for participation is distributed without adequate recognition or support. A full account of participation would therefore include time-to-start, changing support requirements, interruption and re-entry, technical and symptom-related recovery, caregiver or staff support time, and reasons for discontinuation alongside conventional outcomes. A temporally just virtual reality system supports movement while allowing older adults to remain authors of their own pace within justified therapeutic and safety constraints.

## 1 Introduction: the body under timed conditions

Virtual reality (VR) is increasingly used to support physical activity, rehabilitation, and participation in later life (1, 2), but functional gains and engagement capture only part of its value. VR also organizes the temporal conditions of participation—how quickly users must respond, how easily they can pause, and how costly it is to recover from interruptions. That temporal logic is already familiar outside VR. For an older adult with an unsteady gait, the interval between the green figure and the flashing red hand at a pedestrian crossing is not a neutral countdown but a temporal gate, determining how much hesitation is permitted and who must bear the cost of access under conditions of urgency. The problem is not just a slow body but an environment timed for another.

Age-friendly frameworks already recognize that mobility emerges from the relation between individual capacity and environmental conditions (3, 4). Bringing time into that relation makes pace, delay, interruption, and recovery visible. Time is socially organized rather than neutral (5), and institutional timelines can shape what can be asked, observed, sustained, and recognized in human movement research (6). In *Mrs Dalloway*, Virginia Woolf (7) places different lives under a common public clock: the hour is shared but not lived alike. Technology can likewise allocate equal time while imposing unequal temporal demands. In VR, these demands are written into the interface itself: session length, response windows, progression rules, calibration, pause mechanics, and pathways back after interruption shape what counts as timely action, acceptable delay, or legitimate interruption. Participation thus occurs within a temporal order partly produced by the intervention itself.

In this study, VR refers primarily to immersive systems delivered through head-mounted displays (HMDs). The broader VR literature on aging and physical activity has often grouped these systems with semi-immersive environments, 360-degree video, screen-based exergames, and platforms such as Nintendo Wii (8). These systems differ in immersiveness, interaction, physical engagement, and tolerability (8, 9), with consequences for participation in later life (10, 11). Evidence from broader digital or exergaming research is included only when the question at stake does not depend on HMD-specific immersion.

The VR literature reports functional, adherence, acceptability, feasibility, and adverse-event outcomes, but these do not necessarily specify the temporal conditions under which participation occurred—including preparation, support, interruption, and recovery (1, 2, 12). As VR moves from supervised trials to homes and care settings, work performed by researchers or clinical staff may instead fall on users, relatives, or care services (12–14). Such support may be entirely appropriate. What becomes difficult to judge is whether feasibility carries over when the time-work that sustained participation in one setting disappears from view in another.

Exercise, rehabilitation, and technology use inevitably carry temporal demands; effort unfolds in sequence, therapeutic tasks may require cadence, and safety can justify constraints on pace or interruptions. Those demands become a matter of justice when they materially shape who can participate, on what terms, or at whose temporal cost (5, 15). Temporal injustice becomes a concern when such arrangements impose avoidable or insufficiently justified disadvantage; leave foreseeable differences in capacity unaccommodated when reasonable accommodation remains compatible with the intervention’s purpose and safety; or transfer necessary time-work to others without adequate recognition, choice, or support (13, 16). Temporal justice concerns how these demands are justified and how their costs and authority are distributed. I develop the argument through three related analytical dimensions: temporal autonomy, tempo fit, and temporal governance. I also propose a minimum temporal-envelope reporting set to examine what conventional effectiveness and feasibility outcomes can leave unresolved: what participation is required, whose time it requires, and under what conditions it remains possible.

## 2 From dose to tempo fit

Recent studies in human movement research have examined how institutional timelines shape what can be asked, observed, sustained, and recognized (6). The same problem appears on a different scale when a person enters a virtual environment: time is no longer only a condition of research but part of the interaction itself. Drawing on accounts of time as socially organized and unevenly distributed (5, 15), I use three dimensions to examine this temporal organization: temporal autonomy, the extent to which consequential decisions about pace, interruption, progression, and return remain meaningfully available to the user; tempo fit, the relation between the rhythms imposed or invited by the system and the capacities available to the person at that moment; and temporal governance, the way temporal rules, responsibilities, authority, and support work are allocated. The distinctions are analytical; in practice, the three dimensions often converge on the same design feature. A pause function can implicate all three: who can initiate it, whether it accommodates fluctuating capacity, and who determines its conditions and consequences. Figure 1 summarizes these dimensions and their observable implications.

**Figure 1:**
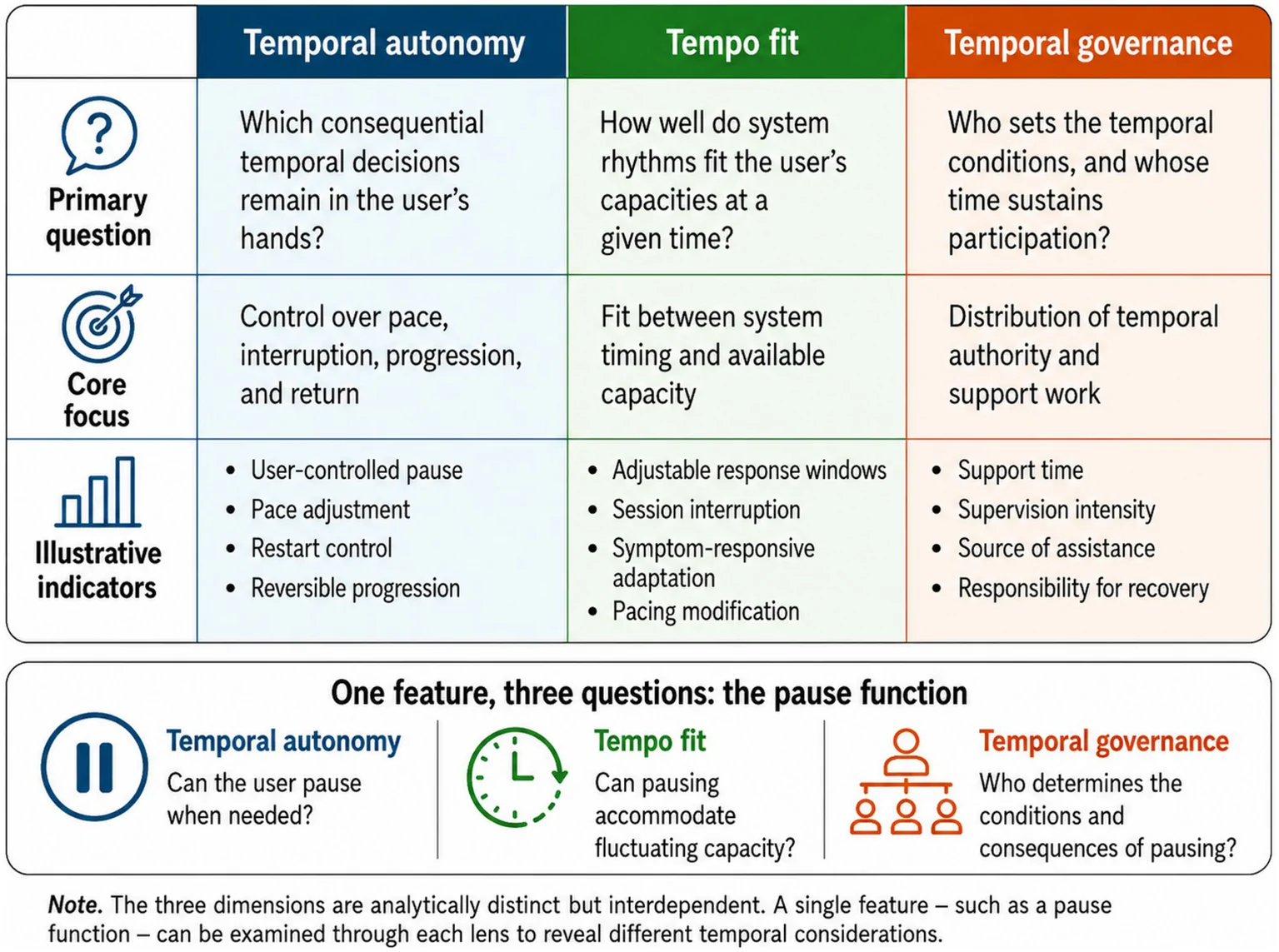
Analytical dimensions of temporal justice in VR.

Temporal justice shares terrain with usability, accessibility, person–environment fit, treatment burden, and implementation research but focuses specifically on how time is organized and distributed within participation. Usability addresses whether technologies can be understood and operated; treatment-burden and implementation frameworks make visible work delegated across patients, social networks, and delivery systems (13, 16, 17). The temporal question is who must adapt to whose pace, which forms of temporal variation the system can accommodate, and whose time becomes necessary when person and system do not readily align. Two systems may be similarly usable while distributing temporal control, support, and time-work very differently. Temporary usability difficulties need not carry implications for temporal justice when adequate support is available.

This distinction predates digital technology. Bergson (18) objected to treating duration as though it were exhausted by homogeneous, interchangeable units. Measurement gives time a common metric; it does not make different durations equivalent to lived conditions. Identical recorded durations can contain very different conditions of participation. The canonical definition of physical activity makes movement measurable and actionable for epidemiology and public health (19), and dose–response evidence remains indispensable (20). Minutes, intensity, expenditure, and adherence tell us how much activity occurred; they say less about the temporal organization that made it possible—the pace required, the possibility of interruption, or the conditions of recovery and return. This becomes particularly consequential in later life because capacities differ between individuals and can also vary within the same person across circumstances. Frailty, for example, can involve reduced strength, slow walking, exhaustion, low physical activity, and weight loss; it is also associated with greater vulnerability to adverse outcomes (21). A temporal demand that is unremarkable under one set of conditions may become difficult after fatigue, illness, pain, poor sleep, or simply on another day.

Tempo fit names that relationship. The same response window may be manageable for one participant, demanding for another, and manageable for the same person on one occasion but not for another. Some tasks depend on cadence, repetition, or progressive challenge. When that rhythm is integral to the therapeutic purpose and safely achievable, preserving it may itself constitute a good temporal fit. Tempo misfit arises when temporal demands and available capacities diverge in ways that materially constrain participation. This relational account is consistent with conceptions of physical activity as situated participation rather than energy expenditure alone (22).

Non-adherence, repeated interruptions, or reliance on support may warrant examination for tempo misfit alongside motivation, preference, prior experience, self-efficacy, socioeconomic circumstances, and other barriers to technology use (23, 24). Such patterns can arise for many reasons: the activity may be uninteresting, symptoms may emerge, equipment may fail, support may be unavailable, or the cause may have little to do with timing. Tempo misfit becomes a defensible interpretation when evidence shows system timing and available capacity are in tension. In VR, timing is implemented through response windows, pause and recovery mechanics, and progression rules, whose consequences depend on both interface and delivery context. Dose and tempo answer different questions. Dose asks how much activity occurred; tempo fit asks what temporal arrangement allowed—or prevented—that activity from occurring as intended. When sustainable participation is the concern, the temporal envelope can help interpret feasibility, adherence, and observed effects without functioning as an intervention outcome in its own right.

## 3 Evidence under tempo: what current studies show—and leave unresolved

Reviews of immersive technologies report favorable findings across some functional, feasibility, acceptability, and user-experience outcomes in older adults, with considerable variation across population, device, protocol, comparator, and delivery context (1). HMD-specific evidence remains heterogeneous. A review of 14 randomized trials involving 839 adults over 60 years found a moderate effect on quality of life, no significant effect on physical activity, low or very low certainty about these outcomes, and limited evidence for pain, perceived effort, and adverse events (2). How commonly VR studies report the temporal conditions of participation remains unknown. The examples below show what conventional outcomes can remain unresolved when those conditions are not described. Part of this heterogeneity lies in the technology itself. Fully immersive HMD systems, semi-immersive environments, exergames, and screen-based tasks differ in immersion, interaction, device configuration, and physical engagement (8). Within HMD-based VR, content, visual stimulation, locomotion, and exposure duration can also influence cybersickness and tolerance (9, 25). The technology through which an intervention is delivered forms part of its temporal conditions of participation.

Feasibility depends on the delivery arrangement. Laboratory and clinical studies may include setup, monitoring, troubleshooting, progression adjustment, and symptom management provided by research or clinical personnel. Yun et al. (26) reported high completion in a therapist-supervised program in which progression was controlled by an occupational therapist; the MOTIVE pilot reported high attendance with interventionist support for setup, technical problems, and safety (12). In a contrasting home-based study, 13 older adults completed 18 self-administered HMD sessions after written guidance and remote familiarization; two of the 15 enrolled participants withdrew because of cybersickness (14). Feasibility is demonstrated within a particular arrangement of training, guidance, supervision, technical assistance, and safety support. Supported delivery may be the appropriate model, but its supporting infrastructure is part of the conditions that make participation possible. When an intervention extends beyond that setting, the work may move with it. A task performed by a researcher or clinician—fitting the headset, resolving a technical failure, adjusting progress, or monitoring symptoms—may become the responsibility of the user, a relative, or a care service.

Similar outcomes can rest on very different arrangements for time and support. VR may remove travel while adding calibration, troubleshooting, safety monitoring, symptom management, or caregiver coordination; conventional delivery may impose scheduling, travel, waiting, or staff demands that VR reduces. What differs is where the necessary time sits, who provides it, and whether it remains available. Adverse effects add another temporal dimension. Cybersickness symptoms such as nausea, dizziness, disorientation, headache, and visual discomfort are well-documented in HMD-based VR (9, 25, 27). Rmadi et al. (28) assessed cybersickness across three HMD sessions in nursing-home residents, while Trillo-Charlín et al. (2) found limited and heterogeneous adverse event evidence. A symptom count or severity score can establish that the symptoms occurred while leaving the subsequent course of participation unclear: whether the session was interrupted, how long recovery took, whether the next exposure changed, or whether the person returned to the intervention at all.

The same gap runs through feasibility, support, and interruption. Session completion does not, by itself, reveal the temporal work through which it was achieved. A temporally informative account would distinguish three linked intervals: entry (setup, calibration, and first interaction), in-session regulation (pacing, pausing, and error recovery), and re-entry (resumption after interruption or between sessions), together with the time-work required across them. These intervals describe the conditions surrounding an outcome rather than constituting a new outcome scale. Recording time-to-start, changing support requirements, interruptions, symptom-related recovery, support time, and discontinuation reasons can help show how feasibility depends on the delivery arrangement in which it was observed. Their interpretation still depends on why time was required, who provided it, and the therapeutic or safety conditions involved. A longer preparation period, repeated pauses, or continuing assistance cannot be judged by duration alone.

## 4 Designing and evaluating for temporal justice

VR interventions embed temporal architectures through defaults, prompts, progression rules, calibration routines, pause mechanics, and recovery pathways. Therapeutic purpose and safety can legitimately constrain intensity, rhythmic cueing, exposure duration, supervision, or interruption. The relevant question is how those constraints are justified, how they operate across differing capacities, and whether they can be reconsidered when needs or circumstances change. A fixed rhythm required by the intervention’s mechanism is not equivalent to a fixed rhythm that is inherited from a software default.

Temporal autonomy and tempo fit become concrete at the interface. A pause function provides a simple example. Its presence alone says little about the temporal control available in practice. Where clinically compatible, users may need opportunities to pause, slow, repeat, stop, and return without avoidable penalties; where adaptation is permitted, response windows, prompt cadence, calibration, comfort settings, and progression can be examined in relation to variation in capacity. A pause function may be difficult to access, result in lost progress, or make returning cumbersome. Control that exists in the interface may remain difficult to exercise in practice. Persuasive systems can shape behavior and engagement, making interruption costs relevant for evaluation (29, 30). HMD tolerability can also vary with exposure duration, visual stimulation, locomotion, content, and individual susceptibility (9, 25, 27).

The same temporal architecture extends beyond the interface into the work required to sustain participation. Setup, device fitting, calibration, updates, troubleshooting, safety monitoring, cleaning, and re-entry may be performed by researchers or clinicians in supported delivery and by users, family members, paid caregivers, or staff elsewhere. Such distributed work is already recognized in treatment-burden and implementation frameworks (13, 16). Temporal governance brings one aspect of that distribution into focus: whose time is required, when it is required, and what happens to participation when that time is unavailable. Home delivery may reduce travel and scheduling demands while introducing additional setup, monitoring, and recovery work. A journey avoided may be replaced by calibration at home; a clinic appointment may disappear while a family member becomes responsible for fitting the device or resolving a technical problem. Where caregiver or staff time is required, claims about scalability depend partly on whether that time remains available, resourced, and incorporated into the delivery model when the intervention moves to another setting. When continuing support is clinically necessary, preferred, or part of the intended delivery model, that support belongs to the intervention rather than sitting outside its description.

This redistribution of time has a public health dimension because the resources needed to absorb temporal demands are unevenly distributed. Digital health literacy among older adults varies with socioeconomic circumstances, device access, place of residence, and social support (31). For VR, affordability, connectivity, domestic space, institutional staffing, and access to paid or unpaid assistance may also shape participation. These conditions affect not only whether a person can obtain or operate a device; they also influence how readily the temporal demands of preparation, troubleshooting, interruption, and recovery can be absorbed. A headset may be available, but the time, space, knowledge, or human support required to use it may not be. The same temporal demand may consequently be minor in a well-supported household or service and prohibitive where assistance is scarce, schedules are inflexible, or technical problems cannot be readily resolved. The consequences depend partly on the resources surrounding the person and on the demands built into the intervention itself.

Co-design can make temporal assumptions contestable before they become fixed in an intervention. Participatory approaches position older adults as contributors to design decisions rather than solely as recipients (32, 33). Applied to VR, co-design can expose assumptions about session length, interruption, progression, recovery, and support through deliberation with users and those who assist them. Its relevance to temporal justice lies in making visible which rhythms are negotiable, which are necessary, whose needs inform them, and how those assumptions hold up when the intervention is used.

VR can extend participation where real environments are difficult to access, but it cannot substitute for making those environments accessible. A virtual park does not solve unsafe pavements, an inadequately timed crossing, or unreliable transport. Virtual access may expand one form of participation while leaving temporal exclusions in the material environment unchanged. Entry time, interruption handling, recovery responsibility, support intensity, and conditions of return can be documented as features of the participation context. Their meaning depends on intervention purpose, safety requirements, individual capacities, and delivery context. Together, they provide empirical material for examining temporal autonomy, tempo fit, and temporal governance (Box 1).

BOX 1 **Minimum temporal-envelope reporting set for VR interventions with older adults**

*Temporal envelope* denotes the observable temporal conditions and time-work surrounding participation, from preparation and entry through interruption, recovery, return, or discontinuation. These variables are descriptive rather than stand-alone indicators of temporal justice and should not be combined into a composite score. When feasibility, transfer across settings, or equity are an issue, the following variables can complement conventional intervention outcomes:

*Time-to-start:* Elapsed time from the first session-specific preparation activity to the start of the planned intervention activity for that session. Where feasible, report component times such as space preparation, device fitting, calibration, controller pairing, updates, login requirements, and orientation. Start and end points should be defined clearly enough to support comparison across sessions and studies and to distinguish demands arising from the technology from those arising from the delivery arrangement.

*Support requirements and trajectory:* Type, intensity, duration, and source of assistance required for participation and how these requirements change through repeated use. Distinguish, where feasible, technical assistance, clinical supervision, passive safety monitoring, equipment preparation, instruction, and recovery or re-entry support. Identify who provides that support—research or healthcare personnel, institutional staff, paid caregivers, or unpaid supporters. Where continuing assistance is clinically necessary, preferred, or built into the delivery model, report it as part of that model.

*Pause and re-entry patterns:* Number and duration of interruptions, classified where possible as voluntary self-regulation, symptom-triggered, technical, safety-related, or externally imposed. Report whether continuity is preserved, whether recalibration or repetition is required, how long meaningful activity takes to resume, and whether an interruption is followed by return.

*Technical interruption and recovery:* Frequency and duration of technical disruptions, the work required to restore participation, who performs that work, and whether the disruption affects session completion or later return.

*Symptom-related interruption and recovery:* Sessions paused, shortened, or stopped because of cybersickness or related symptoms, together with symptom onset, interruption and recovery time, and any effect on subsequent exposure or return. Where feasible, reporting should capture the temporal course of recovery as well as symptom presence or severity.

*Disaggregated reasons for discontinuation:* Where ascertainable, report whether discontinuation was associated with pacing or progression demands, symptoms, technical difficulty, recovery problems, unavailable support, unrelated health events, logistical barriers, loss of interest, or modality preference. Attrition alone does not identify temporal misfit. Any such interpretation requires evidence linking discontinuation to the temporal demands or conditions of participation.

Where possible, situate these observations by device class, setting, and support intensity. The size of the recorded temporal envelope has no fixed evaluative meaning: a small envelope may reflect low requirements, incomplete measurement, participant selection, or brief exposure, while a larger one may reflect necessary and appropriately resourced support. Interpretation should consider intervention purpose, population, safety requirements, delivery model, support preferences, and completeness of reporting.

## 5 Conclusion: time, justice, and the evaluation agenda

Return to the pedestrian crossing. For an older adult with an unsteady gait, timing is a condition of access; VR can reproduce a comparable temporal allocation through pacing, progression, pause, calibration, and re-entry rules. Evidence of effectiveness tells us what changed but leaves part of the participation context out of view. A supervised effect belongs to the delivery arrangement under which it was produced. Whether the same participation remains possible when that arrangement changes is an empirical question.

Temporal justice brings three aspects of that arrangement into view: whether users retain meaningful authority over consequential aspects of pace and interruption; whether system rhythms remain workable in relation to changing capacities; and how the time-work needed to sustain participation is distributed. Prescribed pacing, supervision, and continuing support may be integral to safe and effective delivery. Their temporal significance becomes especially apparent when an intervention moves between settings or when the people and resources that previously sustained participation change. A VR intervention, when demonstrated with substantial staff or caregiver support, may be entirely feasible within that delivery model. Claims about transfer or scalability need to account for what happens when the required time-work is reduced, redistributed, or no longer adequately resourced.

VR can extend participation but cannot substitute for accessible social and built environments. Inside VR, systems can preserve meaningful possibilities for regulating pace, interruption, and return to the extent compatible with therapeutic and safety requirements; outside it, virtual access does not resolve inaccessible streets, transport, or public space. The deeper question is not how much movement VR can produce but whose time that movement is allowed to follow. A body does not move in minutes alone: it hesitates, tires, recovers, interrupts, and begins again. A system may count every minute correctly and still misrecognize the time at which movement becomes possible.

## Data Availability

The original contributions presented in the study are included in the article/supplementary material, further inquiries can be directed to the corresponding author.
